# Supplementary material for: Factors affecting onion production: An empirical study in the Raya Kobo district, Amhara regional state, Ethiopia
Source: PLoS One. 2024 Jun 14;19(6):e0305134. doi: 10.1371/journal.pone.0305134 (PMC11178157; doi:10.1371/journal.pone.0305134)
Supplement: Attachment — Submitted filename: Azhar_Rasool (Response Sheet).pdf [file pone.0305134.s004.pdf]

Journal: PLOS ONE
Manuscript No: PONE-D-24-08353
Title: Factors Affecting Onion Production: an Empirical Study in the Raya Kobo District, Amhara Regional State, Ethiopia

Observation-1: Coherence is lacking among the structured Abstract, Result and discussion, Conclusion and recommendations. Likewise, the author mentioned supply chain in abstract, while value chain and sustainability in conclusion and recommendations. These are different three domains, While the study investigated only factors affecting onion production according to the drawn conceptual framework in the Figure 1

Authors' Response: The authors have revised the manuscript and corrected according to my suggestion in this version.

Observation-2: The author mentioned onion farmers' constraints including pot-harvest handling in section 3.1 aragrapg#3 of results & discussions. While the same problem is not discussed in section 3.4 entitled major constraints of onion farmers in study area.

Authors' Response: The revised manuscript is consistent with the objectives of the study and corrected according to my suggestion.

Observation-3: The study defined about post-harvest losses on section 4. it's not previously discussed anywhere.

Authors' response: The authors deleted the concept of post-harvest loss and revised the manuscript according to the papers' conceptual framework.

Observation-4: According to the defined study, by minimizing the onion farmers' constraints, farmers' wellbeing may improve, crop yield may increase that may impact sectoral growth. But will not stronger the value chain or its' sustainability as defined in the last paragraph.

Authors' response: The authors' updated the manuscript according to my suggestion.

Decision: The authors addressed each comment/observations and the attached the revised manuscript have been **accepted** for further proceedings.
