# Supplementary material for: Factors affecting onion production: An empirical study in the Raya Kobo district, Amhara regional state, Ethiopia
Source: PLoS One. 2024 Jun 14;19(6):e0305134. doi: 10.1371/journal.pone.0305134 (PMC11178157; doi:10.1371/journal.pone.0305134)
Supplement: Attachment — Submitted filename: Review Report.docx [file pone.0305134.s001.docx]

Factors Affecting Onion Production: an Empirical Study in the Raya Kobo District,
Amhara Regional State, Ethiopia

**Reviewer comments:**

The overall structure of the paper needs improvement. The abstract part is shallow and lacks the research gap and others. The introduction does not provide a clear roadmap for the study, and the flow of ideas throughout the paper is not well organized.

The methodology section lacks sufficient detail on the data collection process, sample size, and sampling techniques. It's important to provide a clear understanding of how the data was gathered and analyzed.

The choice of statistical methods should be justified more thoroughly. Were there any alternative methods considered, and if so, why were they not chosen?

The paper lacks a discussion on data quality and potential biases in the dataset. It's crucial to assess the reliability of the data and discuss any limitations that might affect the study's findings.

The theoretical literature review is essential to provide a more comprehensive overview of the existing literature, including recent developments in the field.

The paper introduces a theoretical framework, but it's not well-integrated with the research questions or hypotheses. The authors should clarify how the theoretical framework guides the study.

The results are presented in a somewhat fragmented manner. The authors should consider reorganizing them to make the findings more coherent and easier to follow.

The discussion of results is relatively brief and lacks critical analysis. The authors should investigate deeper into the implications of their findings and relate them to the existing literature.

The conclusion is somewhat abrupt and doesn't provide a robust summary of the paper's main contributions and their implications for policy and practice. A more detailed discussion of policy recommendations is needed.

The paper contains several grammatical and stylistic errors. The authors should carefully proofread and edit the manuscript for clarity and consistency.

These comments should help the authors improve their paper by addressing key aspects of clarity, abstract, introduction, methodology, literature review, theoretical framework, results, discussion, recommendation, and overall presentation.
