# Supplementary material for: Factors affecting onion production: An empirical study in the Raya Kobo district, Amhara regional state, Ethiopia
Source: PLoS One. 2024 Jun 14;19(6):e0305134. doi: 10.1371/journal.pone.0305134 (PMC11178157; doi:10.1371/journal.pone.0305134)
Supplement: Attachment — Submitted filename: review response onion pone.docx [file pone.0305134.s003.docx]

Journal: PLOS ONE

Manuscript No: PONE-D-24-08353

Title: Factors Affecting Onion Production: an Empirical Study in the Raya Kobo District, Amhara Regional State, Ethiopia

The authors are thankful to reviewers for their comments to improve the quality of our manuscript.

Please find below our response to the each of the comments in the order in which they have been raised.

**Reviewer 1:**
The overall structure of the paper needs improvement. The abstract part is shallow and lacks the research gap and others. The introduction does not provide a clear roadmap for the study, and the flow of ideas throughout the paper is not well organized.

**Response:** Thank you for your comments. We have rewrite and revised it accordingly.

The methodology section lacks sufficient detail on the data collection process, sample size, and sampling techniques. It's important to provide a clear understanding of how the data was gathered and analyzed.

The choice of statistical methods should be justified more thoroughly. Were there any alternative methods considered, and if so, why were they not chosen?

**Response:** Thank you for your comments. We have revised the data description section and provided justification for the chosen statistical methods of analysis in this version.

The paper lacks a discussion on data quality and potential biases in the dataset. It's crucial to assess the reliability of the data and discuss any limitations that might affect the study's findings.

**Response:** Thank you for your comments. We have added the discussion of data quality checks in this version and pointed that there are no limitations that affect the findings of the study.

The theoretical literature review is essential to provide a more comprehensive overview of the existing literature, including recent developments in the field. The paper introduces a theoretical framework, but it's not well-integrated with the research questions or hypotheses. The authors should clarify how the theoretical framework guides the study.

**Response:** Thank you for your comments. We have tried to review the theoretical and empirical literatures on the area, and we have build our own framework that are well integrated with the research hypothesis.

The results are presented in a somewhat fragmented manner. The authors should consider reorganizing them to make the findings more coherent and easier to follow.

**Response:** Thank you for your comments. We have revised and corrected it.

The discussion of results is relatively brief and lacks critical analysis. The authors should investigate deeper into the implications of their findings and relate them to the existing literature.

**Response:** Thank you for your comments. We have revised and cross-checked with the existing literatures in this version of the manuscript.

The conclusion is somewhat abrupt and doesn't provide a robust summary of the paper's main contributions and their implications for policy and practice. A more detailed discussion of policy recommendations is needed.

**Response:** Thank you for your comments. We have revised and corrected it.

The paper contains several grammatical and stylistic errors. The authors should carefully proofread and edit the manuscript for clarity and consistency.

**Response:** Thank you for your comments. We have tried to check the grammatical and stylistic errors in this version as much as possible.

These comments should help the authors improve their paper by addressing key aspects of clarity, abstract, introduction, methodology, literature review, theoretical framework, results, discussion, recommendation, and overall presentation.

**Response:** Thank you for your comments. We have revised all the sections of the manuscript according to your suggestion in this version.

**Reviewer 2:**
Coherence is lacking among the structured Abstract, Result and discussion, Conclusion and recommendations. Likewise, the author mentioned supply chain in abstract, while value chain and sustainability in conclusion and recommendations. These are different three domains, While the study investigated only factors affecting onion production according to the drawn conceptual framework in the Figure 1.

**Response:** Thank you for your comments. We have revised and corrected according to your suggestion in this version.

Secondly, author mentioned onion farmers' constraints including pot-harvest handling in section 3.1 paragrapg#3 of results & discussions. While the same problem is not discussed in section 3.4 entitled major constraints of onion farmers in study area.

**Response:** Thank you for your comments. We have revised and corrected according to your suggestion in this version- making consistent to the objectives of the study.

Thirdly, study defined about post-harvest losses on section 4. it's not previously discussed anywhere.

**Response:** Thank you for your comments. We have revised and deleted the post harvest loss concepts as the paper focuses only on the determinants of onion production in this version.

Fourthly, according to the defined study, by minimizing the onion farmers' constraints, farmers' well-being may improve, crop yield may increase that may impact sectoral growth. But will not stronger the value chain or its' sustainability as defined in the last paragraph.

**Response:** Thank you for your comments. We have revised and corrected according to your suggestion in this version.
