# Supplementary material for: Factors affecting onion production: An empirical study in the Raya Kobo district, Amhara regional state, Ethiopia
Source: PLoS One. 2024 Jun 14;19(6):e0305134. doi: 10.1371/journal.pone.0305134 (PMC11178157; doi:10.1371/journal.pone.0305134)
Supplement: Attachment — Submitted filename: Factors affecting onion production.docx [file pone.0305134.s006.docx]

Factors affecting onion production: an empirical study in the Raya Kobo district, Amhara regional state, Ethiopia

The authors address most of the issues raised. But, still it needs some modifications (like abstract part, the study area, and others have been given comments on manuscripts).
